# Spatial Distribution of Flower Color Induced by Interspecific Sexual Interaction

**DOI:** 10.1371/journal.pone.0164381

**Published:** 2016-10-10

**Authors:** Yuma Takahashi, Koh-ichi Takakura, Masakado Kawata

**Affiliations:** 1 Frontier Research Institute for Interdisciplinary Sciences, Tohoku University, 6–3, Aoba, Aramaki, Aoba, Sendai, Miyagi, 980–8578, Japan; 2 Division of Ecology and Evolutionary Biology, Graduate School of Life Sciences, Tohoku University, 6–3, Aoba, Aramaki, Aoba, Sendai, Miyagi, 980–8578, Japan; 3 School of Environmental Science, University of Shiga Prefecture, 2500, Hassaka-cho, Hikone, Shiga, 522–8533, Japan; Institute of Genetics and Developmental Biology Chinese Academy of Sciences, CHINA

## Abstract

Understanding the mechanisms shaping the spatiotemporal distribution of species has long been a central concern of ecology and evolutionary biology. Contemporary patterns of plant assemblies suggest that sexual interactions among species, i.e., reproductive interference, lead to the exclusive distributions of closely related species that share pollinators. However, the fitness consequences and the initial ecological/evolutionary responses to reproductive interference remain unclear in nature, since reproductive isolation or allopatric distribution has already been achieved in the natural community. In Japan, three species of blue-eyed grasses (*Sisyrinchium*) with incomplete reproductive isolation have recently colonized and occur sympatrically. Two of them are monomorphic with white flowers, whereas the other exhibits heritable color polymorphism (white and purple morphs). Here we investigated the effects of the presence of two monomorphic species on the distribution and reproductive success of color morphs. The frequency and reproductive success of white morphs decreased in area where monomorphic species were abundant, while those of purple morphs did not. The rate of hybridization between species was higher in white morphs than in the purple ones. Resource competition and habitat preference seemed not to contribute to the spatial distribution and reproductive success of two morphs. Our results supported that color-dependent reproductive interference determines the distribution of flower color polymorphism in a habitat, implying ecological sorting promoted by pollinator-mediated reproductive interference. Our study helps us to understand the evolution and spatial structure of flower color in a community.

## Introduction

Elucidating the factors that affect the spatiotemporal variations of species assemblies has long been a central concern of ecology. Biotic interactions, as well as historical and phylogenetic factors, play a role in shaping biodiversity gradients throughout species turnover because of resource competition and niche segregation and/or reproductive isolation after secondary contact [1]. For flowering plants, some studies have shown that flowers of co-occurring species to be more dissimilar than expected by chance [2–4]. The distribution of floral traits in communities is expected to exhibit greater variance than expected by random assembly from a regional species pool [1]. Negative interactions between species that share pollinators are suggested to influence the structure of community and spatial distribution of flower color [5,6].

The fitness cost of interspecific sexual interactions has been observed in many plant and animal taxa and is generally described as reproductive interference [7]. Reproductive interference can be observed in various events in the process of reproduction. In plants, hetero-specific pollens deposited on stigmas by interspecific pollen transfer sometimes reduce the seed set of recipient species [8,9]. Theoretical and empirical studies suggest that reproductive interference has an exceptionally strong influence on ecological dynamics compared with resource competition [7,10]. This is because reproductive interference can more readily result in competitive exclusion than ordinary resource competition because of its positive frequency dependence [7,11] and its self-reinforcing impact via positive feedback [7,12]. Recently, exclusive distributions of closely related species were studied in various systems and could be explained by reproductive interference [1]. However, fitness consequence and the initial process of evolutionary/ecological responses of reproductive interference at relatively short timescales, such as spatial variation in traits or morph/allele frequency within a habitat and the reinforcement of the reproductive isolation, remain unclear in the natural condition. Generally, because each species should achieve sufficient reproductive isolation or allopatric distribution in both ecological and evolutionary time scale, it is difficult to demonstrate the fitness responses and its ecological and evolutionary consequences of reproductive interference in natural settings, particularly in species assemblies of native species.

It is also difficult to identify the mechanisms shaping species assemblies, since the difference in the resource competition between species as well as reproductive interference should simultaneously affect the reproductive success of each species and the consequence of inter-specific interactions. However, we can infer the relative importance of reproductive interference and resource competition. Importantly, if resource competition predominant, individual fitness of given two species should depend on total density rather than frequency of the two species [13]. In contrast, individual fitness tended to depend on the relative abundance (frequency) of the two species, whenever reproductive interference is major factor affecting spatial distribution of the species.

**Fig 1:**
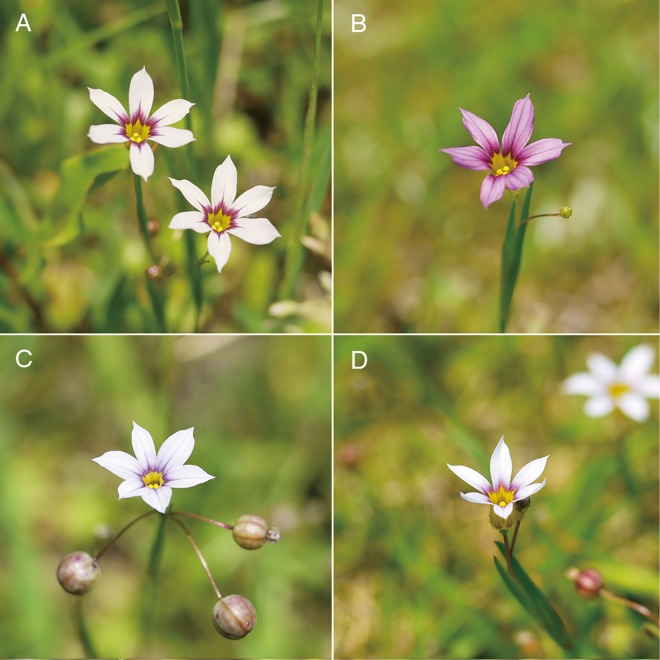
The three species of *Sisyrinchium*. The M-species exhibits two morphs consisting of a white (a) and purple morph (b). L-species (c) and S-species (d) are monomorphic (only white). L-species is taller and has bigger fruits than M- and S-species. S-species has smaller flower than that of other species. Flowers of L- and S-species are more constricted at the middle of the tepals than M-species. Photographs were taken by YT.

In Japan, three morphologically distinct species of *Sisyrinchium* have been independently colonized since the late 1800s via human activity probably from different geographic regions in South America [14]. Three species were identified as different species, but the scientific names have not been formally assigned due to taxonomic confusion. The three species expanded rapidly throughout Japan and often occur sympatrically [15], indicating that they experienced contemporary artificial secondary contact in Japan. The large size species (L-species) and small size species (S-species) are monomorphic with white flowers (Fig 1). On the other hand, middle size species (M-species), which is the most common, exhibits heritable flower color polymorphism (white and purple morphs) within a population (Fig 1). The flower color morphs are controlled by two alleles at a single autosomal locus [16]. Although hybrid individuals between these three sympatric species are very rare [16], they could interact with each other via inter-species pollen transfer during the daily blooming period in a natural setting. This means that the system with three species, one of which has genetic color polymorphism, can provide us an opportunity to test the initial process and evolutionary consequences of reproductive interference. In addition, comparison between color morphs for the ecological and evolutionary responses to the presence of closely related species enable to us to more easily find the contribution of reproductive interference to species assemblies, since color morphs are expected to have similar ecological traits such as resources competition ability.

Here we aim to demonstrate the effect of reproductive interaction between the M-species and the two other species of *Sisyrinchium* on their reproductive success and the spatial variation of morph frequencies of the M-species in the natural populations, assuming that morph frequencies have reached an evolutionary equilibrium in each population [17]. First, we compared the morphology, floral color, spatial distribution, and daily blooming rhythm of the three species to reveal the possibility of reproductive interference among them. Second, we investigated the relationship between the relative abundance of the two species (L- and S-species) and the distribution of the two color morphs of the M-species in their natural habitats. Third, we asked whether the reproductive success of the two color morphs of the M-species changed depending on the relative abundance of the S- and L-species. Our study provides some evidence supporting ecological sorting promoted by pollinator-mediated reproductive interference.

Potentially, in nature, not only morph-specific reproductive interference but also morph-specific ecological traits such as competition ability for resources and pollinators and habitat preferences can generate the spatial variations of color morph frequency. For example, in *Linanthus parryae*, the difference in the preference for soil moisture between morphs contributed to the establishment of spatial variation in morph frequency in habitat [18]. To eliminate the possibilities of other than reproductive interference as mentioned above, the effects of the density (a total number of individuals of the three species) and the distance from water’s edge on morph frequency and reproductive success were analyzed. Since the distance from water’s edge may be correlated with soil environment such as soil moisture, the significant effect of the distance from water’s edge on the morph frequency would be detected when the difference in habitat preference between morphs contributes to morph frequency variation [18]. If there were differences in competition ability for resources and pollinators between morphs, we would find the differential fitness responses between morphs against the total density of the three species, because a competition is generally density-dependent [13]. On the other hand, we would find the differential fitness response between morphs against the relative abundance (frequency) of other species, whenever reproductive interference is major factor affecting the reproductive success and thus spatial variation of color morphs. Note that, depending on the mechanisms of reproductive interference, differential fitness responses between morphs may be found against both frequency and density.

## Materials and Methods

### Study system

*Sisyrinchium* (Iridaceae: Iridoideae) is a remarkably diverse and widespread genera in Iridaceae. Almost all species are native to the Americans. Particularly in the *S*. *micranthum* species group, including *S*. *micranthum*, *S*. *laxum*, and *S*. *rosulatum*, the wide range of intraspecific variation in morphology and floral color has been reported within and among populations [19]. The taxonomy of *S*. *micranthum* has long been misunderstood [20]. In Japan, three morphologically and genetically (S1 Fig) distinct *Sisyrinchium* species have been independently colonized since the late 1800s and often occur sympatrically [15,16]. In the present study, we refer to these three species as large (L-), middle (M-), and small (S-) species. Traditionally, these three species are identified as *S*. *atlanticum* in Japan, although morphological and phylogenetic considerations are not enough. However, morphologically, it is clear that these three species are included in the *S*. *micranthum* species group that includes *S*. *micranthum* and *S*. *roslatum*, rather than *S*. *atlanticum* species group, which belongs to a distantly related group of *Sisyrinchium*, as suggested by Yamaguchi & Hirai [16]. Indeed, the nucleotide sequence of the internal transcribed spacer region suggests that all three species are included in the *S*. *micranthum* group (S1 Fig), though the three species do not share the same haplotype even when they coexist. In the present study, we tentatively used “*Sisyrinchium* spp.” for the three species because of the lack of taxonomic resolution [14]. Both outbreeding and self-fertilization can occur in all each species [16].

All three species mainly occur open turf around a pond or a fen. The flowering period is usually from May to June. As in the case of native habitat in South America [21], bees and hover flies visit the flowers of this species (Y. Takahashi, personal observations). Flower longevity is generally one day, during which flower anthesis is typically restricted around noon. Although the L- and S-species are monomorphic with white flowers, the M-species exhibits discrete, heritable flower color polymorphism (white and purple morphs) within a population. The two color morphs sympatrically distribute in a habitat and they do not seem to show ecological preference [16]. The flower color morphs of the M-species are controlled by two alleles (*H*^*W*^ and *H*^*P*^) at a single autosomal locus; the purple morph is homozygous recessive (*H*^*P*^*H*^*P*^) and the white morph is heterozygous and homozygous dominant (*H*^*W*^*H*^*P*^, *H*^*W*^*H*^*W*^) [16]. The two morphs of the M-species may be maintained by overdominant selection within a population [17].

The M- and L-species sometimes produce hybrids, in which seed parents seem to always be the L-species [16], though no hybrid individuals between L- and S-species and M- and S-species have been found. Hybrid individuals between M- and L-species are almost always sterile; they do not develop healthy pollens or fruits [16]. This finding was supported by molecular analysis (S1 Fig). Hybrids between the white morph of the M- and L-species can be distinguished from those hybrids between the purple morph of the M- and L-species on the basis of flower color [16]. The former crossings produce hybrids with a light-pink flower, whereas the latter crossings produce hybrids with a light-violet flower (S2 Fig), meaning that we can identify the paternal morph of hybrid individuals depending on their flower color.

### Study sites

Fieldwork was conducted in 15 sites in Japan (S1 Table). All the turfs studied were located adjacent to rivers or ponds. The distribution of flowers at each site was uniform or random rather than patchy, suggesting that enough time to expand their distribution in the habitat had elapsed since their initial invasion. Though species composition in the turfs studied was not same among the sites, the environmental condition of the turf was qualitatively similar to each other. For all of these locations, no specific permissions were required since our sampling did not involve endangered or protected species.

### Morphological measurements

We collected individuals of the S-, M-, and L-species and hybrids between the M- and L-species from five habitats, where multiple species co-occur (Ibusuki, Shiga, Toyohashi, Sagara and Tsuchiura). The number of individuals of each species/morph tested was as follow, Ibusuki: S-species [S]: 24, purple morph of M-species [M_P_]: 22, white morph of M-species [M_W_], L-species [L]: 13, light-violet morph of hybrid [Hv]: 15; Shiga: M_P_: 5, M_W_: 5; Toyohashi: S: 3; Sagara: S: 7; Tsuchiura: Hv: 7, light-pink morph of hybrid [H_P_]: 7). For all individuals, the height, flower diameter, length and width of the outer and inner tepals, length of the highest internode, length of the peduncle, and length of highest leaf were measured immediately after collection. Principal component analysis (PCA) was conducted to extract useful and independent variables from the multivariate data sets. PCA detected two effective dimensions (PC1 and PC2) that explained 89.0% of the total variance. PC1 corresponded to body size, and PC2 corresponded to relative flower size. Differences among categories (species and morph) for each principal component were analyzed with the Tukey HSD test.

To estimate the possibility that insect pollinators would discriminate between different flowers, the reflectance spectrum of the flower (nectar guide and tepal) and background environment were measured with a spectrometer (USB2000; Ocean Optics, Inc., Dunedin, FL, USA). The background color was estimated by averaging the reflectance spectra measured from green leaves of the three species. The illuminant D65, which is a commonly used standard illuminant defined by the International Commission on Illumination (http://www.cis.rit.edu/research/mcsl2/online/cie.php), was used as reference for the daylight spectra. Spectral reflectance measurements covered the range 300–700 nm (interval: 1.0 nm). These spectra were used for calculating the color loci of the targets in the space of honeybee color vision [22]. Although discrimination ability varies with the situation, approximately 0.1 color distance in hexagon units is required for approximately 60% correct discrimination in bees [23]. The number of individuals for each species/morph measured was S: 10, M_P_: 19, M_W_, 12 and L: 21 for nectar guides, and S: 11, M_P_: 16, M_W_, 13 and L: 25 for tepals.

### Spatiotemporal distribution and reproductive success

In order to investigate the spatial distribution and the daily blooming rhythm of the three species, 1 m^2^ quadrats were laid out in a grid pattern at 5 m intervals on turf adjacent to a pond in Ibusuki in 9th May, 2013, where all the three species and color morphs occurred. At each quadrat, the number of flowers of each species and color morph was recorded every hour from 6:00 to 16:00.

To reveal the relationship between morph frequency of the M-species and the relative abundance of the other species (the number of individuals of S- and L-species in a quadrat divided by the total number of individuals of all species in a quadrat), quadrat samplings, where quadrats were laid out in a grid pattern at >5 m intervals on the turf adjacent to a pond or fen as far as possible in 13 populations in 2011 and/or 2013 (Otto [2011], Toyama [2011], and Tsukuba [2011], Otaka [2011], Tsuchiura [2011 and 2013], Otaka-pond [2011 and 2013], Shiga [2013], Ibusuki [2013], Sendai [2013], Hirose [2013], Kawaguchi [2013], Banpaku [2013]). Mean number of quadrats in each population in each year is 32 (minimum: 12, maximum: 82). In addition, the number of hybrid individuals was recorded for each quadrat in Tsuchiura, where hybrid individuals were more abundant than in other populations. To estimate the equilibrium frequency of purple morphs when both the L- and S-species were absent, two GLMs, i.e., a simple GLM and a GLM weighted by the sample size of the M-species, were performed for all populations. In both the models, the relative abundance of the L- and S- species was used as an explanatory variable and a binomial distribution was assumed for the frequency of purple morphs. The inverse-logits of the y-intercept based on the GLMs were calculated as an estimated frequency of purple morphs when both the L- and S-species were absent.

In six populations (Otaka, Tsuchiura, Nagoya, Banpaku, Otto, and Ibusuki) where the census was conducted, the number of fruits, *F*, the number of peduncles without both a flower and a fruit, *P*, and the diameter of mature fruits, *D*, in a flowering shoot were recorded for individuals of the M-species sampled randomly in a quadrat in 2013. Mean number of quadrats in each population in each year is 18 (minimum: 6, maximum: 32). The fruit set rate and the reproductive success of a single flower was calculated as *F*/(*F* + *P*) and *F*/(*F* + *P*) × *D*, respectively. For the M-species, the number of seeds in a fruit is known to strongly correlate with the diameter of the fruits, irrespective of color morph [17]. The reproductive success was analyzed as an index of reproductive output, i.e., individual fitness. To infer the proximate factors affecting reproductive success, the responses of fruit set rate and the diameter of the fruits against the presence of congeneric species was analyzed independently.

### Statistical analysis

All statistical analyses were performed using R version 3.1.1 [24]. Statistical significance was determined at the level *P* = 0.05. Error bars are SE of mean values. Morph frequencies derived from multiple populations over a couple of years were analyzed by generalized linear model (GLM) assuming binomial error distribution. The relative abundance of the L- and S-species, population and year were used as explanatory factors. The diameter of mature fruits in a flowering shoot, the fruit set rate and the reproductive success derived from multiple populations was analyzed by GLM assuming Gaussian or binomial error distribution. The relative abundance of the L- and S-species, total density, population were used as explanatory factors. Levels of significance were calculated using the R package “car” [25].

## Results

### Spatiotemporal distribution of three species

**Fig 2:**
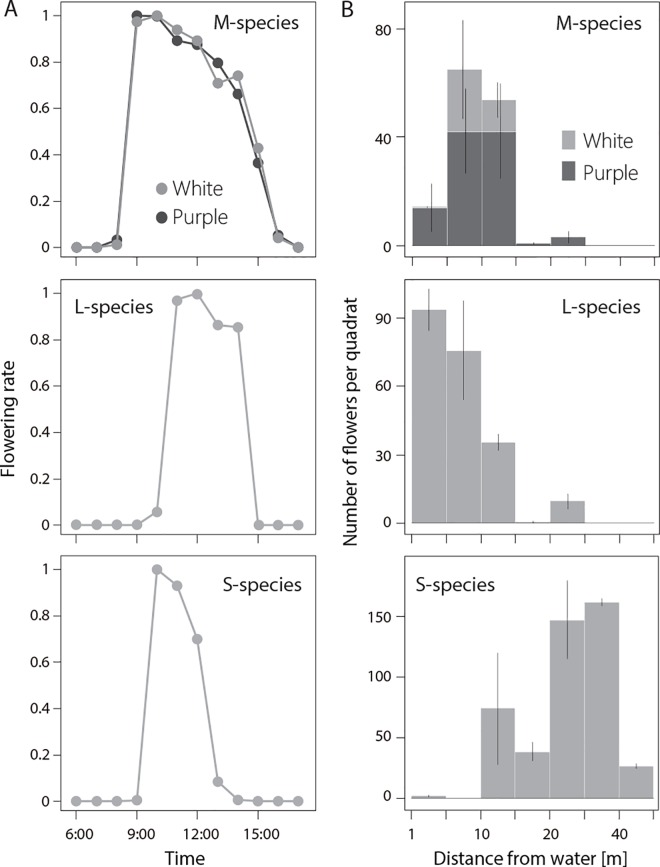
**Diurnal blooming rhythm (a) and spatial distribution of the three species (b).** Top, middle, and bottom panels represent the results for M-, L-, and S-species, respectively. Error bars are SE.

The diurnal changes in flowering rates (the number of flowers in each hourly census divided by the maximum number of flowers in hourly census for each species) were similar for all species (Fig 2A). Although the flowering period of the M-species was longer than that of the two other species, flowering rates peaked around noon for all species. Total number of flowers of each species/morph observed at peak hour of each species/morph was 1532 for S-species, 459 for purple morph of M-species, 147 for white morph of M-species, and 883 for L-species.

The census during 11:00–12:00 showed that the flowers of the L-species were mainly found near the water, whereas those of the S-species were found the area away from water (Fig 2B). Both color morphs of the M-species were abundant in between the areas occupied by the L- and S-species. Although they tended to be exclusive, there were some areas of overlap.

### Morphological analysis

**Fig 3:**
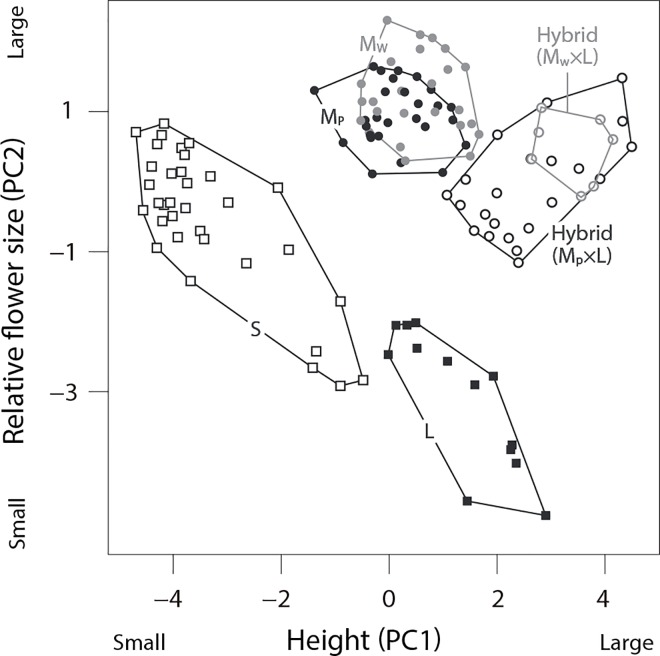
The results of the principal component analysis on morphological traits of the three species and the hybrid between the M- and L-species. PC1 and PC2 were mainly characterized by body size and relative flower size, respectively. M_P_: purple morph of M-species, M_W_: white morph of M-species, S: S-species, L: L-species. Total number of individuals of each species/morph tested is as follow; S: 34, M_P_: 27, M_W_:25, L: 13, hybrid (M_P_×L): 22, hybrid (M_W_×L): 7.

Although morphological variations within a species (particularly in height) were large in the L- and S-species compared to color morphs of the M-species and hybrids, no overlaps among the three species were found in principal component analysis (PCA) space (Fig 3) (see also S2 Table). Both PC1 and PC2 varied significantly among six categories (ANOVA: PC1, *F* = 149.34, *P* < 0.001; PC2, *F* = 69.153, *P* < 0.001). A post-hoc comparison detected significant differences between species in either PC1 or PC2 for all species, whereas significant differences were not found between color morphs in both the M-species and hybrids.

**Fig 4:**
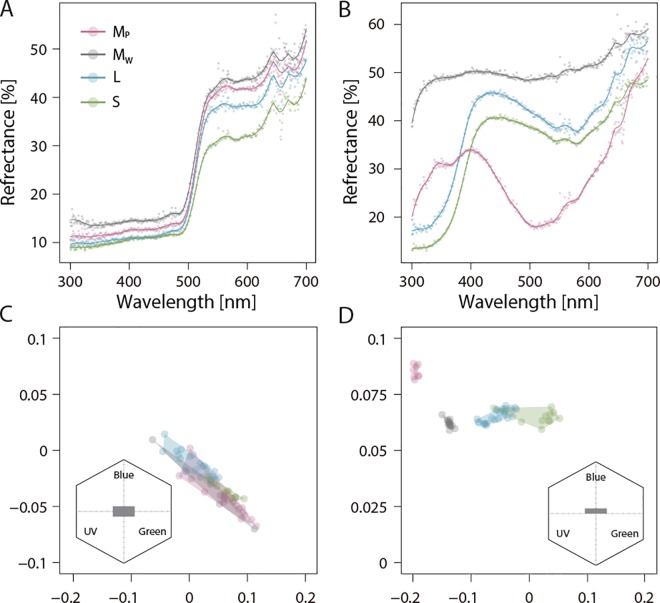
**Mean spectral reflectance spectrum of the nectar guide (a) and tepal (b) of the three species, and the corresponding color loci plotted (c: nectar guide, d: tepal) in a hexagon color space.** Areas indicated by gray box in the inset plot were enlarged for detail (d, c).

The patterns of reflectance spectra of the nectar guides were not so different among species and morphs, though mean values varied among species probably because of differences in the thickness of tepals, because background color could influence the value of reflectance especially when tissue measured is thin or transparent (Fig 4A). Reflectance spectra of the tepals were different among species and morphs (Fig 4B). The nectar guide colors in the eyes of bees were overlapped in hexagon color space for all morphs and species (Fig 4C), whereas significant variations among categories (species and morphs) were observed for both x- (*F* = 11.463, *P* < 0.0001) and y-coordinates (*F* = 18.54, *P* < 0.0001). Although statistical significance was found in some combinations between species for the *x*- and *y*-coordinates in the post-hoc comparisons, the mean color distances were less than 0.1 in hexagon units for all combinations. On the other hand, tepal colors in the eyes of bees significantly varied among categories (species and morphs) with less overlapped in hexagon color space (x-coordinate, *F* = 364.71, *P* < 0.0001; y-coordinate (*F* = 196.72, *P* < 0.0001) (Fig 4D). Post-hoc comparisons found significant differences in all combinations between categories for the x- and/or y-coordinates in hexagon color space, whereas all except the purple morph of the M-species tended to clump together. A mean color distance greater than 0.1 in hexagon units was found between the L-species and the purple morphs of the M-species and between the S-species and the two morphs of the M-species.

### Color morph frequency

**Fig 5:**
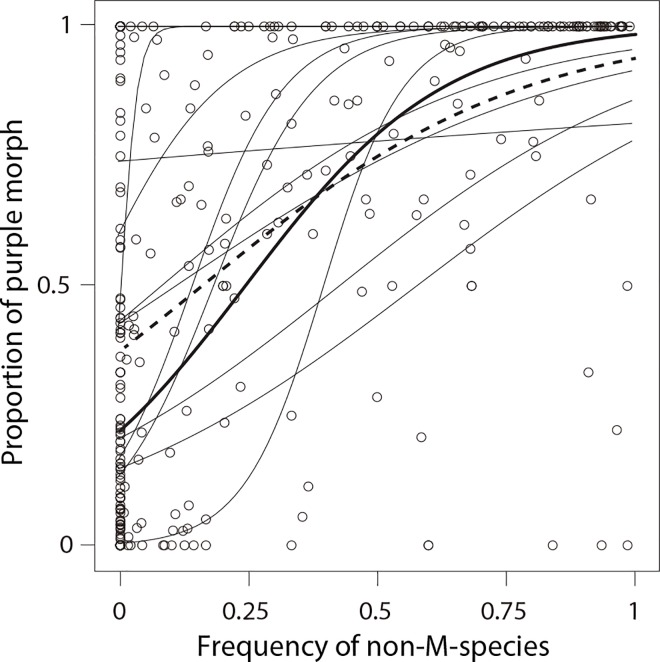
Relationship between the relative abundance of L- and S-species in the *Sisyrinchium* community and the proportion of purple morphs. Thin lines are logistic regression lines for each study population. The bold line and the dashed line indicate the weighted and non-weighted regression lines for all populations except the monomorphic populations.

Both purple and white color morphs were found in all populations except in the Tsukuba and Kawaguchi populations (S1 Table). In populations where multiple species coexisted, frequency of the purple morphs of the M-species in each quadrat significantly increased with the relative abundance of the L- and S-species (Fig 5, relative abundance of the L- and S-species [A]: *χ*^2^ = 1132.1, *P* < 0.001; population [Pop]: *χ*^2^ = 5017.8 12, *P* < 0.001; year: *χ*^2^ = 0.0, *P* = 0.979; A×Pop: *χ*^2^ = 501.2, *P* < 0.001). That is, most of the quadrats where the white morph was at high frequency lie in the area between the other two species, while most of the quadrats where the white morph is at low frequency occur where the other two species were common.

In most of populations, where only L- and M-species occurred, the morph frequency showed clinal variation along the distance from water’s edge; the proportion of purple morphs was higher at sites adjacent to the water’s edge dominated by the L-species than sites distant from water. As a result, the relative abundance of L-species was strongly correlated with the distance from water’s edge (distance: *χ*^2^ = 21.01, *P* < 0.001; population: *χ*^2^ = 324.76 *P* < 0.001; Year: *χ*^2^ = 0.86, *P* = 0.355), meaning that we cannot include the two factors into a same statistical model to find the contribution of them due to multicollinearity. To avoid such statistical problem, we used populations where three species occurred (Ibusuki, Tsukuba), where site adjacent to the water’s edge and the distance from water’s edge were dominated by the L- and S-species, respectively. In these populations, the relative abundance of non-M-species (L- and S-species) was not correlated with the distance from water’s edge (distance: *χ*^2^ = 0.0127, *P* = 0.91; population: *χ*^2^ = 6.727, *P* < 0.01; year: *χ*^2^ = 0.1171, *P* = 0.732), and then non significant effect of distance from water’s edge on color morph frequency was found while the effect of the relative abundance of non-M-species are was significant (relative abundance of the L- and S-species: *χ*^2^ = 77.099, *P* <0.001; distance: *χ*^2^ = 0.418, *P* = 0.518; population: *χ*^2^ = 24.99, *P* < 0.001; year: *χ*^2^ = 19.252, *P* < 0.001). A similar exclusive distribution pattern was found in the census along the water’s edge, where the abiotic environment was relatively constant (S3 Fig).

Equilibrium frequency of purple morphs when both the L- and S-species were absent (Y-intercept of the regression line in Fig 5) was estimated at 0.287 ± 0.004 (mean ± SE) and 0.218 ± 0.0004 (mean ± SE) in non-weighted and weighted least square regression, respectively.

### Inter-specie sexual interaction

Spatial distribution of the two morphs in hybrids was examined in the Tsuchiura population. The frequency of the light-violet morphs in hybrid individuals, of which the paternal morph is the purple morph of the M-species, was significantly lower than the frequency of purple morphs in the M-species (S4 Fig, M-species or hybrid: *χ*^2^ = 22.08, *P* < 0.001; distance from the water’s edge: *χ*^2^ = 12.927, *P* < 0.001; interaction: *χ*^2^ = 0.033, *P* = 0.855), suggesting higher hybridization rate in white morphs of M-species than purple ones.

**Fig 6:**
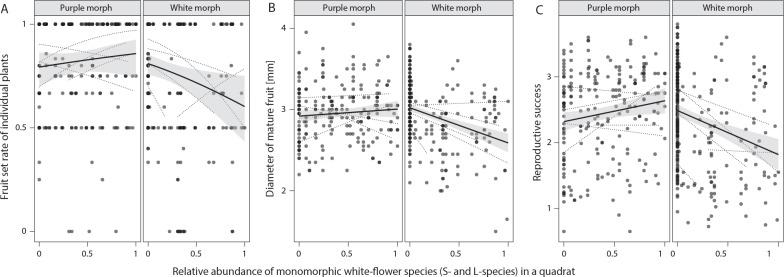
**Fruit set rate (a), the diameter of mature fruit (b), and the female reproductive success (c) of two color morphs of M-species as a function of the relative abundance of monomorphic white-flower species (S- and L-species) in a quadrat.** Dotted lines are regression line for each population used. Bold regression lines are estimated by regression for all data points without any regard to populations. The shaded area indicates the confidence interval of the regression lines.

The fruit set rate of the white morphs of the M-species decreased with increasing in relative abundance of monomorphic white-flower species (the L- and S-species) in a same quadrat, but that of the purple morphs did not (Fig 6). A significant interaction effect between the morph and the relative abundance of the white-flower species was detected while non-significant interaction effect between the morph and the total density of three species (relative abundance of the white-flower species [A]: *χ*^2^ = 1.428, *P* = 0.232; density [D]: *χ*^2^ = 0.616, *P* = 0.433; morph [M]: *χ*^2^ = 13.90, *P* < 0.001; population: *χ*^2^ = 32.721; *P* < 0.001, A×D: *χ*^2^ = 1.643, *P* = 0.20; A×M: *χ*^2^ = 6.793, *P* = 0.009; D×M: *χ*^2^ = 0.056, *P* = 0.813). Fruit diameter, which is an index of the number of seeds within a fruit, decreased with the relative abundance of the white-flower species only in the white morphs. Significant interaction effects between the morph and the relative abundance of the white-flower species was detected (relative abundance of the white-flower species [A]: *χ*^2^ = 7.947, *P* = 0.005; density [D]: *χ*^2^ = 1.401, *P* = 0.237; morph [M]: *χ*^2^ = 15.534, *P* < 0.001; population: *χ*^2^ = 75.68, *P* < 0.001; A×D: *χ*^2^ = 0.057, *P* = 0.812; A×M: *χ*^2^ = 11.478, *P* < 0.001; D×M: *χ*^2^ = 3.322, *P* = 0.068). Consequently, the reproductive success of the white morphs of the M-species decreased with the relative abundance of the white-flower species, though that of purple morphs was not affected by the abundance of congeneric species. Reaction of the reproductive success against the relative abundance of the white-flower species was different between morphs, but that against density was not (relative abundance of the white-flower species [A]: *χ*^2^ = 2.973, *P* = 0.085; density [D]: *χ*^2^ = 0.496, *P* = 0.481; morph [M]: *χ*^2^ = 12.50, *P* < 0.001; population: *χ*^2^ = 74.75, *P* < 0.001; A×D: *χ*^2^ = 0.075, *P* = 0.784; A×M: *χ*^2^ = 13.54, *P* < 0.001; D×M: *χ*^2^ = 0.240, *P* = 0.624).

## Discussion

Microevolutionary investigations of intraspecific variation in floral traits are important for identifying the role of adaptive evolution in floral diversification. We focused on the effect of interspecific reproductive interaction among congeneric species on the distribution of color morphs of M-species of *Sisyrinchium*. The morphologies of the three sympatric species were discontinuous. Though the differentiation in microhabitat environments might partly contribute the morphological differences, this fact suggests genetic differentiation and isolation among species, since the distribution of three species partly overlapped in their habitats. Nearly synchronous flowering and overlap distribution imply the possibility of reproductive interaction among three congeneric species. In the present study, we demonstrated that the white morph frequency of the M-species decreased with the relative abundance of the other species with similar flower color (L- and S-species), suggesting an exclusive relationship between the white morph of the M-species and the other species. The reduction of reproductive success in the presence of white flower species was observed in the white morphs, but not in purple ones. As discussed in detail below, our findings suggest that color-dependent interspecific reproductive interaction shapes the spatial pattern of color polymorphism in the M-species of *Sisyrinchium* sp., i.e., ecological sorting promoted by pollinator-mediated reproductive interference. This study may help our understanding of the evolution of floral color and spatial distribution of flower color in a community.

Though we did not qualitatively investigate the pollinator community of *Sisyrinchium* spp., small bees were often observed visiting and foraging the flowers during our census. In the present study, analysis of flower color suggests that it is harder for the bees to distinguish flowers of the white morphs from those of other species, compared with purple morphs, assuming they have similar visual literacy to honey bees. Insect pollinators often sequentially visit one flower type (so called flower constancy), even when many other flowers are available [6,26]. Therefore, the white morphs of the M-species and other species (L- and S-species) might share pollinators, which results in pollen exchanges between species with them. Indeed, the frequency of the blue morphs in hybrid individuals was lower than the frequency of purple morphs in the M-species in a natural population, implying that the rate of hybridization between the L-species and the white morphs of the M-species is higher than that between the L-species and the purple morphs of the M-species. In addition, reproductive success of M-species decreased only in white morphs when the relative abundance of other species was high. Such color-specific responses suggest that flower constancy of pollinators increase pollen exchange among multiple species having similar floral traits, and thus leads a color-dependent reproductive interference, possibly shaping the spatial pattern of color polymorphism in the M-species of *Sisyrinchium*.

Reproductive interference in plants can be caused by pollen transfer between different congeneric species by pollinators [10,27,28]. In our system, inter-species pollen transfer by bees might increase the rate of production of inviable hybrids and reduce the rate of conspecific fertilization in the white morphs of the M-species, thus reducing their fruit set rate and the number of seeds within a fruit. At this time, however, we cannot completely rule out the possibility that inter-morph physiological differences in interspecific pollen rejection resulted in morph specific fitness reduction when occurring with different species. Future studies, including artificial pollination, behavioral observation of pollinators, transplantation experiments and observation of pollen dynamics on a stigma, are needed to confirm the ecological or physiological mechanism of the morph specific cost of reproductive interference between congeneric species.

For flowering plants, the negative effects of reproductive interference among species that share pollinators may potentially influence community assembly [2]. In *Pedicularis*, pollinator-mediated reproductive interference promotes ecological sorting on a large geographic scale [1]. Because differences in floral traits reduce the transfer of heterospecific pollen [29,30], co-occurring species exhibit significantly greater variance or dissimilarity in floral traits [2]. In the present study, we found that the recurring spatial variation of floral color within a population was a microevolutionary consequence of reproductive interference. The increase in the frequency of purple morphs in a habitat where they co-occur with L- and S-species can also be interpreted as the process of the reinforcement of reproductive isolation of the M-species. Our findings may contribute to understand the process of the evolution of floral color in a community, as well as mechanisms that shape the spatial pattern of flower color within and among populations, thus community assembly.

Morph frequency variation that we observed in the present study can be potentially explained by differential habitat preference, such as differential water use, and differential competition ability between color morphs. However, exclusive distribution between the white morph of the M-species and the other species was also observed in the census along the water’s edge, where soil condition (e.g., soil moisture) is expected to be relatively constant, and no significant effect of the distance from the water’s edge on morph frequency was found in the natural populations. These results imply no potential difference in habitat preference between morphs. Moreover, we did not find any significant evidence supporting the contribution of differences in competition ability (interaction effect of morph and total density) on reproductive success. Spatial pattern of flower color polymorphism in the natural habitats can be mostly explained by color-dependent reproductive interference rather than difference in competition ability and/or habitat preference, though we do not have any direct evidence at this time.

In the present study, we found smooth clinal variation in morph frequency in along the distance from the water’s edge, where L-species is abundant. Theoretically, the combination of divergent selection along the environmental continuum and balancing selection acting on morphs is suggested to result in spatial cline in allele or morph frequency [31–33]. Actually, in M-species, overdominance has been demonstrated by artificial pollination experiments [17]. In addition, the frequency of purple morphs in the M-species that occurred without other species was similar to the frequency predicted by overdominant selection (25% purple phenotypes), suggesting the presence of overdominant selection for two color morphs. This implies that equilibrium frequency of color morph in M-species in nature is determined by the cooperation of balancing selection and directional selection derived from color-dependent reproductive interference. Since three *Sisyrinchium* species has slightly different habitat preference, clinal variation in morph frequency of the M-species may be explained by the combination of overdominant selection and the spatial variation in the strength of directional selection determined by the abundance of congeneric species. In general, gene flow within a population is one of factors establishing smooth clinal variation in morph frequency along the environmental gradient in a habitat [34]. However, the shape and position of clinal variation in morph frequency is not affected by gene flow as long as balancing selection is acting [32].

Our results cannot explain the inter-population variation in the clinal pattern of morph frequency in the M-species along the relative abundance of other species. This variation may be because of the difference in the period of co-habitation of the three species; the morph frequency cline in some populations may have not reached an evolutionary equilibrium state [1]. Other unknown factors, such as differences in the community of pollinators, herbivores and competitors, may contribute to the inter-population variation. In addition, the equilibrium frequency of color morphs of the M-species may be affected not only by the relative abundance of congeneric species compared to the M-species but also by the density of the M-species, suggesting the presence of unknown factors affecting the relative frequency and thus morph frequency of the M-species (S5 Fig). Further studies assessing the ecological and historical factors that affect morph frequency may also help us understand the establishment of species assembly and community structure.

## Supporting Information

S1 Fig Phylogenetic relationship of three speciess and the outgroup, *Sisyrinchium atlanticum*, constructed by Neighbor-joining method, based on 572 bp of ITS region (Accession number: LC055683–LC055720). Branch numbers represent percentage of bootstrap values in 1050 sampling replicates and the scales indicate branch length. The words following species name represent population names (S1 Table). Population Kajiki locates at 31.7227°N, 130.6495°E.
(DOCX)

S2 Fig Frequency of purple morphs around hybrid individuals. The number of purple and white morphs of M-species was counted in quadrat where either morph of hybrid individuals was centered. Photographs were taken by Y.T.
(DOCX)

S3 Fig Relationship between the relative abundance of L-species and the frequency of purple morphs of M-species found in quadrat sampling along the water's edge. Solid line is the logistic regression line weighted by sample size of M-species.
(DOCX)

S4 Fig Morph frequencies in M-species (*a*) and hybrid (*b*) along the distance from the pond in Tsuchiura population. The frequency of purple morphs in M-species was significantly higher than the frequency of light-violet morphs, which are produced by the crossing between purple morph of M-species and L-species.
(DOCX)

S5 Fig Effect of density and the relative abundance of non-M-species on the frequency of purple morph of M-species. Both the density of non-M-species and relative abundance of non-M-species affected morph frequency of M-species. Generalized additive model found significant effect of the relative abundance (*χ*^2^ = 91.33, *P* < 0.001) and the density of non-M-species (*χ*^2^ = 16.30, *P* < 0.001) on the frequency of purple morph of M-species.
(DOCX)

S1 Table Study sites and sample size for each analysis. (DOCX)

S2 Table Summary of morphological data (mm). (DOCX)
