# Supplementary material for: Spatial Distribution of Flower Color Induced by Interspecific Sexual Interaction
Source: PLoS One. 2016 Oct 10;11(10):e0164381. doi: 10.1371/journal.pone.0164381 (PMC5056732; doi:10.1371/journal.pone.0164381)
Supplement: S1 Fig — Branch numbers represent percentage of bootstrap values in 1050 sampling replicates and the scales indicate branch length. The words following species name represent population names (S1 Table). Population Kajiki locates at 31.7227°N, 130.6495°E. (DOCX) [file pone.0164381.s001.docx]

**S1 Fig.** **Phylogenetic relationship of three species and the outgroup, *Sisyrinchium atlanticum*, constructed by Neighbor-joining method, based on 572 bp of ITS region (Accession number: LC055683–LC055720).** Branch numbers represent percentage of bootstrap values in 1050 sampling replicates and the scales indicate branch length. The words following species name represent population names (see Table S1). Population Kajiki locates at 31.7227°N, 130.6495°E.
