# Supplementary material for: Spatial Distribution of Flower Color Induced by Interspecific Sexual Interaction
Source: PLoS One. 2016 Oct 10;11(10):e0164381. doi: 10.1371/journal.pone.0164381 (PMC5056732; doi:10.1371/journal.pone.0164381)
Supplement: S2 Fig — The number of purple and white morphs of M-species was counted in quadrat where either morph of hybrid individuals was centered. Photographs were taken by Y.T. (DOCX) [file pone.0164381.s002.docx]

**S2 Fig. Frequency of purple morphs around hybrid individuals.** The number of purple and white morphs of M-species was counted in quadrat where either morph of hybrid individuals was centered. Photographs were taken by Y.T.
