# Supplementary material for: Spatial Distribution of Flower Color Induced by Interspecific Sexual Interaction
Source: PLoS One. 2016 Oct 10;11(10):e0164381. doi: 10.1371/journal.pone.0164381 (PMC5056732; doi:10.1371/journal.pone.0164381)
Supplement: S3 Fig — Solid line is the logistic regression line weighted by sample size of M-species. (DOCX) [file pone.0164381.s003.docx]

**S3 Fig. Relationship between the relative abundance of L -species and the frequency of purple morphs of M-species found in quadrat sampling along the water's edge.** Solid line is the logistic regression line weighted by sample size of M-species.
