# Supplementary material for: Spatial Distribution of Flower Color Induced by Interspecific Sexual Interaction
Source: PLoS One. 2016 Oct 10;11(10):e0164381. doi: 10.1371/journal.pone.0164381 (PMC5056732; doi:10.1371/journal.pone.0164381)
Supplement: S4 Fig — The frequency of purple morphs in M-species was significantly higher than the frequency of light-violet morphs, which are produced by the crossing between purple morph of M-species and L-species. (DOCX) [file pone.0164381.s004.docx]

**S4 Fig**. **Morph frequencies in M-species (*a*) and hybrid (*b*) along the distance from the pond in Tsuchiura population.** The frequency of purple morphs in M-species was significantly higher than the frequency of light-violet morphs, which are produced by the crossing between purple morph of M-species and L-species.
