# Supplementary material for: Spatial Distribution of Flower Color Induced by Interspecific Sexual Interaction
Source: PLoS One. 2016 Oct 10;11(10):e0164381. doi: 10.1371/journal.pone.0164381 (PMC5056732; doi:10.1371/journal.pone.0164381)
Supplement: S5 Fig — Both the density of non-M-species and relative abundance of non-M-species affected morph frequency of M-species. Generalized additive model found significant effect of the relative abundance (χ2 = 91.33, P < 0.001) and the density of non-M-species (χ2 = 16.30, P < 0.001) on the frequency of purple morph of M-species. (DOCX) [file pone.0164381.s005.docx]

**S5 Fig. Effect of density and the relative abundance of non-M-species on the frequency of purple morph of M-species.** Both the density of non-M-species and relative abundance of non-M-species affected morph frequency of M-species. Generalized additive model found significant effect of the relative abundance (*χ*^2^ = 91.33, *P* < 0.001) and the density of non-M-species (*χ*^2^ = 16.30, *P* < 0.001) on the frequency of purple morph of M-species.
