# Supplementary material for: Spatial Distribution of Flower Color Induced by Interspecific Sexual Interaction
Source: PLoS One. 2016 Oct 10;11(10):e0164381. doi: 10.1371/journal.pone.0164381 (PMC5056732; doi:10.1371/journal.pone.0164381)
Supplement: S1 Table — (DOCX) [file pone.0164381.s006.docx]

**S1 Table. Study sites and sample size for each analysis.**

| Location | Latitude | Longitude | Number of samples for distribution analyses  (S-, M-, L-species) | Number of samples for fitness analyses  (purple, white morph) |
| --- | --- | --- | --- | --- |
| Ibusuki | 31.256076 | 130.653007 | 1424, 547, 860 | 39, 45 |
| Shiga | 35.260613 | 136.218516 | 1582, 5898, 150 | ­– |
| Nagoya | 35.154648 | 136.964827 | – | 75, 74 |
| Toyohashi | 34.770701 | 137.396011 | – | – |
| Sagara | 34.829187 | 137.273254 | – | – |
| Tsuchiura | 36.061800 | 140.221348 | 0, 755, 800 | 58, 55 |
| Sendai | 38.223238 | 140.873048 | 0, 1046, 0 | – |
| Hirose | 38.242063 | 140.885165 | 0, 69, 455 | – |
| Otaka-pond | 35.067403 | 136.958394 | 143, 313, 423 | – |
| Otaka | 35.065670 | 136.959080 | 0, 715, 141 | 21, 24 |
| Kawaguchi | 36.104389 | 140.008738 | 0, 822, 11 | – |
| Banpaku | 36.062267 | 140.071751 | 0, 332, 390 | 47, 17 |
| Otto | 36.041956 | 140.148497 | 0, 1966, 6 | 0, 125 |
| Toyama | 36.782245 | 137.105707 | 0, 2305, 29 | – |
| Tsukuba | 36.157542 | 140.062645 | 0, 443, 428 | – |
