# Supplementary material for: Spatial Distribution of Flower Color Induced by Interspecific Sexual Interaction
Source: PLoS One. 2016 Oct 10;11(10):e0164381. doi: 10.1371/journal.pone.0164381 (PMC5056732; doi:10.1371/journal.pone.0164381)
Supplement: S2 Table — (DOCX) [file pone.0164381.s007.docx]

**S2 Table. Summary of morphological measurements (mm).**

| Trait | Species/morph | *N* | Mean | SD |
| --- | --- | --- | --- | --- |
| Height | L-species | 13 | 252.7 | 58.3 |
|  | S-species | 34 | 54.9 | 28.5 |
|  | M-species (purple morph) | 27 | 105.9 | 14.4 |
|  | M-species (white morph) | 25 | 102.1 | 19.0 |
|  | Hybrid (light violet morph) | 22 | 181.3 | 34.5 |
|  | Hybrid (light pink morph) | 7 | 160.0 | 19.1 |
| Flower diameter | L-species | 13 | 12.1 | 0.8 |
|  | S-species | 34 | 9.5 | 1.2 |
|  | M-species (purple morph) | 27 | 15.8 | 1.1 |
|  | M-species (white morph) | 25 | 16.1 | 1.2 |
|  | Hybrid (light violet morph) | 22 | 16.1 | 1.4 |
|  | Hybrid (light pink morph) | 7 | 18.0 | 1.4 |
| Inner tepal width | L-species | 13 | 2.5 | 0.3 |
|  | S-species | 34 | 1.9 | 0.2 |
|  | M-species (purple morph) | 27 | 3.1 | 0.3 |
|  | M-species (white morph) | 25 | 3.2 | 0.2 |
|  | Hybrid (light violet morph) | 22 | 3.6 | 0.4 |
|  | Hybrid (light pink morph) | 7 | 3.4 | 0.2 |
| Inner tepal length | L-species | 13 | 10.0 | 0.3 |
|  | S-species | 34 | 8.0 | 0.7 |
|  | M-species (purple morph) | 27 | 10.7 | 0.6 |
|  | M-species (white morph) | 25 | 11.1 | 0.7 |
|  | Hybrid (light violet) | 22 | 11.8 | 1.2 |
|  | Hybrid (light pink) | 7 | 12.8 | 1.7 |
| Outer tepal width | L-species | 13 | 3.1 | 0.3 |
|  | S-species | 34 | 2.3 | 0.3 |
|  | M-species (purple morph) | 27 | 3.6 | 0.3 |
|  | M-species (white morph) | 25 | 3.7 | 0.3 |
|  | Hybrid (light violet morph) | 22 | 4.2 | 0.4 |
|  | Hybrid (light pink morph) | 7 | 4.4 | 0.2 |
| Outer tepal length | L-species | 13 | 10.8 | 0.4 |
|  | S-species | 34 | 8.3 | 0.8 |
|  | M-species (purple morph) | 27 | 11.3 | 0.6 |
|  | M-species (white morph) | 25 | 11.9 | 0.7 |
|  | Hybrid (light violet morph) | 22 | 12.7 | 1.5 |
|  | Hybrid (light pink morph) | 7 | 14.7 | 0.6 |
| Peduncle length | L-species | 13 | 33.1 | 2.5 |
|  | S-species | 34 | 18.9 | 2.9 |
|  | M-species (purple morph) | 27 | 22.0 | 1.2 |
|  | M-species (white morph) | 25 | 22.9 | 2.1 |
|  | Hybrid (light violet morph) | 22 | 29.4 | 2.7 |
|  | Hybrid (light pink morph) | 7 | 31.9 | 3.1 |
| Highest internode | L-species | 13 | 98.9 | 16.5 |
|  | S-species | 34 | 41.8 | 19.7 |
|  | M-species (purple morph) | 27 | 48.8 | 9.5 |
|  | M-species (white morph) | 25 | 47.5 | 10.4 |
|  | Hybrid (light violet morph) | 22 | 76.0 | 7.1 |
|  | Hybrid (light pink morph) | 7 | 72.3 | 8.2 |
| Highest leaf length | L-species | 13 | 27.5 | 3.9 |
|  | S-species | 34 | 19.9 | 6.0 |
|  | M-species (purple morph) | 27 | 20.8 | 2.1 |
|  | M-species (white morph) | 25 | 20.4 | 2.9 |
|  | Hybrid (light violet morph) | 22 | 24.3 | 1.5 |
|  | Hybrid (light pink morph) | 7 | 24.5 | 1.6 |
